# Venous Response to Tourniquet Pressure in Children: Implications for Peripheral Intravenous Access

**DOI:** 10.1111/apa.70567

**Published:** 2026-04-30

**Authors:** Karl Rönnegård, Staffan Eksborg, Pedram Hosseiniakram, Per‐Arne Lönnqvist, Dmitry Grishenkov, Gunilla Lööf

**Affiliations:** ^1^ Department of Physiology and Pharmacology Karolinska Institutet Solna Sweden; ^2^ Department of Paediatric Anaesthesia and Intensive Care Karolinska University Hospital Solna Sweden; ^3^ Department of Women's and Children's Health Karolinska Institutet Solna Sweden; ^4^ Department of Biomedical Engineering and Health Systems KTH Royal Institute of Technology Stockholm Sweden

**Keywords:** child, peripheral catheterization, tourniquet, vein diameter, venous compliance

## Abstract

**Aim:**

To investigate the impact of different tourniquet pressures and application durations on venous diameter and compliance in children, in order to optimise conditions for peripheral intravenous catheter insertion.

**Methods:**

This non‐randomised clinical study included 21 healthy children aged 6–10 years. Vein diameter was measured using ultrasound. The force required to fully compress the vein was measured with a pressure‐sensing device. Tourniquet pressures of 20–100 mmHg were applied for 60 s, with measurements every 10 s. Data were analyzed using non‐parametric methods to assess changes over time and correlations between variables.

**Results:**

Vein diameter changed significantly over time at all applied pressures. All tourniquet pressures showed a significant reduction in venous compliance. Substantial interindividual variation was observed in the pressure and timing that yielded optimal changes in vein diameter and compliance. Vein diameters ranged from 1 to 5 mm, with no consistent correlation with age or body weight.

**Conclusion:**

Paediatric tourniquet application markedly increases venous diameter and reduces venous compliance. The substantial interindividual variation in these responses motivates individualised rather than universal approaches. Future research should aim to develop practical techniques to assess and optimise venous conditions, thereby improving the success rate of paediatric intravenous catheter insertion.

## Summary

- Optimising venous conditions is essential for successful intravenous access in children, yet evidence on the effects of tourniquet pressure and duration is lacking.
- All applied pressures significantly increased vein diameter and reduced venous compliance, with marked interindividual variation in optimal response.
- These findings highlight the need for individualised tourniquet application and further research to develop practical techniques to assess and optimise venous conditions.

**Abbreviation**
PIVC: peripheral intravenous catheter

## 1 Introduction

Peripheral intravenous catheter (PIVC) insertion is one of the most commonly performed procedures in paediatric healthcare. Despite being a routine procedure, the success rate of PIVC insertion in children remains low. Fewer than 50% of insertions succeed on the first attempt, and in some cases the procedure fails entirely [1, 2]. This often necessitates multiple needle insertions, contributing to significant physical and psychological distress for the child [1, 2, 3] and psychological distress for caregivers [2] and healthcare staff [4], as well as increased healthcare costs [5].

The application of a tourniquet is routinely used to increase venous diameter and reduce compliance, thereby facilitating venipuncture and intravenous access. They may be used either without pressure control (non‐pneumatic elastic devices) or with controlled pressure through pneumatic devices equipped with manometers, typically similar to those used for blood pressure measurement. By increasing venous diameter and reducing venous compliance, tourniquet application facilitates venipuncture and intravenous access. However, the optimal pressure and duration of application remain uncertain.

While studies in healthy adult volunteers indicate that tourniquet pressure influences both venous size and compliance [6], with venous dimensions also varying with application time [7], evidence in paediatric populations is limited, highlighting the need for age‐specific clinical guidelines.

The present non‐randomised clinical study aimed to examine the effects of tourniquet pressure and application duration on venous size and compliance in children. The overarching goal was to identify parameters that may optimise PIVC insertion and support evidence‐based practice in paediatric care.

## 2 Participants and Methods

Following ethical approval from the Swedish Ethical Review Authority (Dnr: 2023‐03064‐01), children were recruited from a local primary school. All children who met the inclusion criteria of being essentially healthy and aged 6–10 years were enrolled in the study (*n* = 21). The sample size was guided by previous studies in adults with a similar experimental design, where this number of participants was sufficient to address the primary research questions.

Oral and written information was provided, and written informed consent was obtained from both children and guardians prior to inclusion.

### 2.1 Equipment

#### 2.1.1 Ultrasound

A point‐of‐care ultrasound system (GE Healthcare, Chicago, IL, USA) equipped with a high‐frequency linear transducer (L10‐22).

#### 2.1.2 Cuff Inflator System

Tourniquet pressure was applied using an MX550 monitor (Philips Healthcare, Best, Netherlands) with pediatric‐sized cuffs.

#### 2.1.3 Pressure Sensor

Venous compliance was measured with a pressure‐sensing device developed in collaboration with the KTH Royal Institute of Technology, Stockholm, Sweden [8]. The system is a research prototype developed and calibrated within this project and has been experimentally tested in a clinical‐like setting.

### 2.2 Setting

All examinations were conducted in temperature‐controlled hospital facilities. The environment was maintained within typical indoor hospital conditions (approximately 20°C–22°C).

Participants were positioned in a supine position with a slightly elevated head. The left arm was placed on an integrated hand rest. A flexible support arm was mounted on the bed in order to minimise unintended ultrasound transducer cable movement. A pressure cuff was selected based on the upper arm circumference, according to the manufacturer's instructions. Using ultrasound, the largest identifiable superficial vein within the left antecubital fossa was selected for measurement. The target vein was typically the cephalic or median cubital vein. In one case, only the basilic vein was suitable for examination.

The transducer position was bordered with thick foam tape to ensure consistent assessment of the same venous site and a consistent location for the repeated application of force. This created both a marking of the site and a physical barrier that prevented unintended transducer displacement. After applying ultrasound transmission gel, the transducer was placed with minimal pressure.

Tourniquet inflation, imaging, and pressure recording were synchronised using a metronome signalling 10‐s intervals. Each instance of pressure was applied carefully with the ultrasound transducer, ensuring that the compression was just sufficient to collapse the vein. Vein diameter and the force required for complete compression were measured every 10 s for one minute. The procedure was conducted both without tourniquet pressure and with tourniquet application at pressures of 20, 40, 60, 80, and 100 mmHg. Inflation to the target pressure occurred within approximately 3–4 s across the pressure range used in the study.

All ultrasound examinations were performed by the first author, an experienced clinician in ultrasound‐guided peripheral venous cannulation in paediatric patients.

**FIGURE 1:**
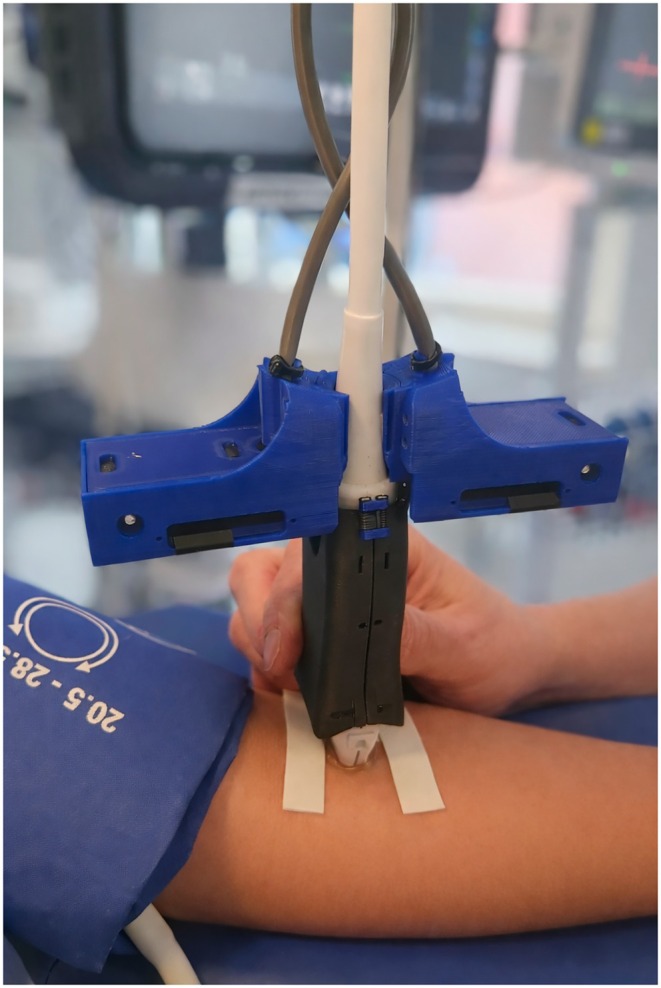
Measurement setup.

The measurement setup is illustrated in Figure 1.

### 2.3 Statistical Analysis

The venous response to tourniquet application was analysed using the Friedman test with Dunn's multiple comparison test to identify statistically significant changes over time. The Wilcoxon matched‐pairs signed rank test was used to compare variation in compliance and diameter. Spearman's rank correlation was used to assess the association between variables.

Statistics were calculated using version 5.04 of GraphPad Prism (GraphPad Software, San Diego, CA, USA). Two‐sided *p*‐values of < 0.05 were considered statistically significant.

## 3 Results

A total of 21 children (11 boys and 10 girls with a median age of 8 years) met the inclusion criteria and were enrolled in the study. Isolated pressure sensor time points were missing for some participants. Consequently, these participants (*n* = 10) were excluded from analyses requiring complete time‐dependent pressure sensor data. All other analyses were performed using the available data, as they relied on aggregated median values rather than complete time‐point data. No data were missing for the vein diameter measurements.

**FIGURE 2:**
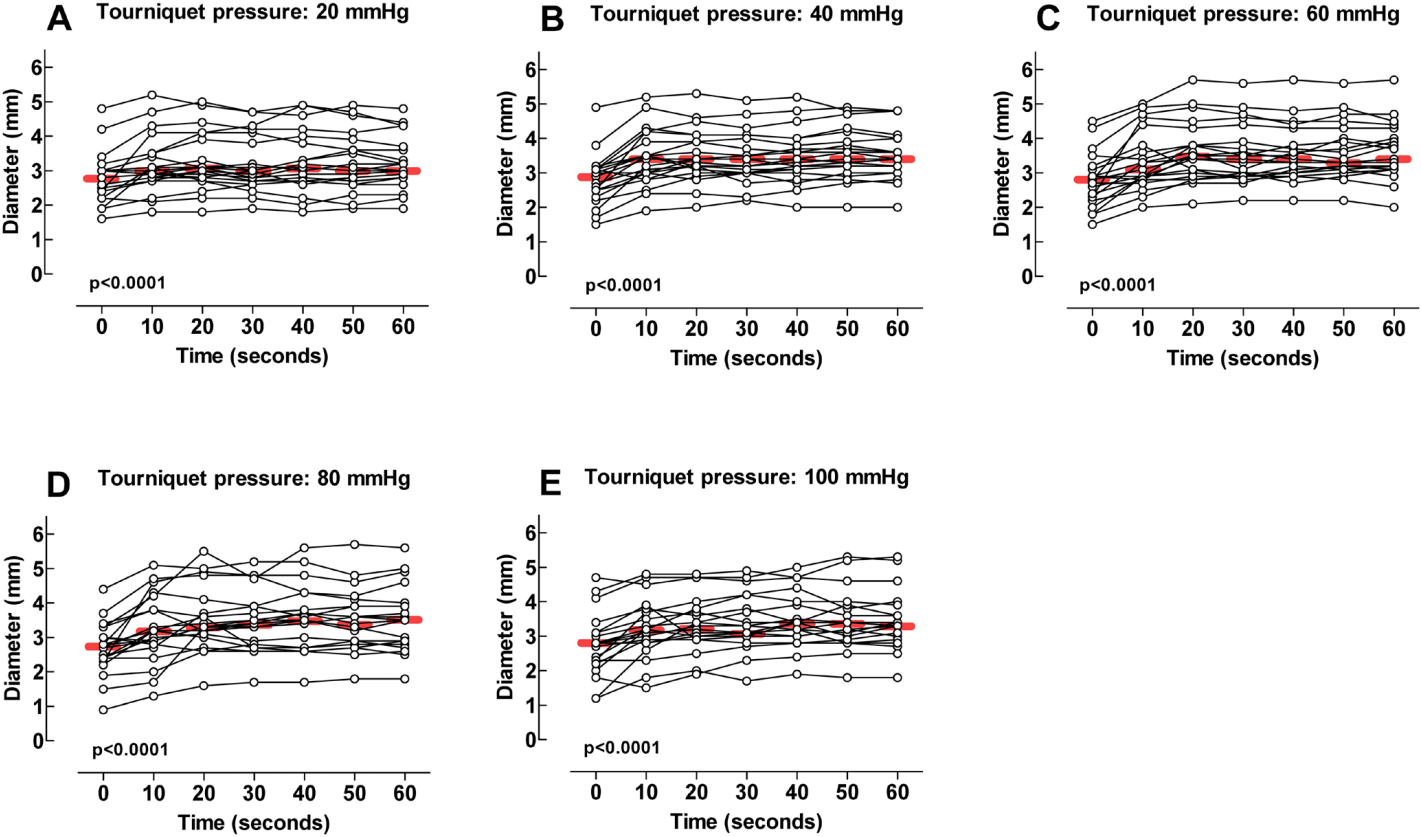
Change in individual and group median vein diameter over 60 s of tourniquet application at 20, 40, 60, 80, and 100 mmHg. The red lines indicate the median values of the diameters at the various time points.

Individual and group median time‐dependent changes in vein diameter after applying tourniquet pressures of 20–100 mmHg are presented in Figure 2. At all pressures, vein diameter increased significantly compared with baseline (*p* < 0.0001). The dilation occurred early after tourniquet application, typically within the first 20 s, after which no statistically significant or consistent changes were observed. At the group level, comparisons across pressure levels showed that vein diameter increased between the lower and intermediate ranges but did not increase further at higher pressures. However, this group pattern was accompanied by substantial interindividual variability in both magnitude and timing, and was therefore not representative of all participants. Individual time‐dependent changes in vein diameter for all participants are presented in Table S1.

**FIGURE 3:**
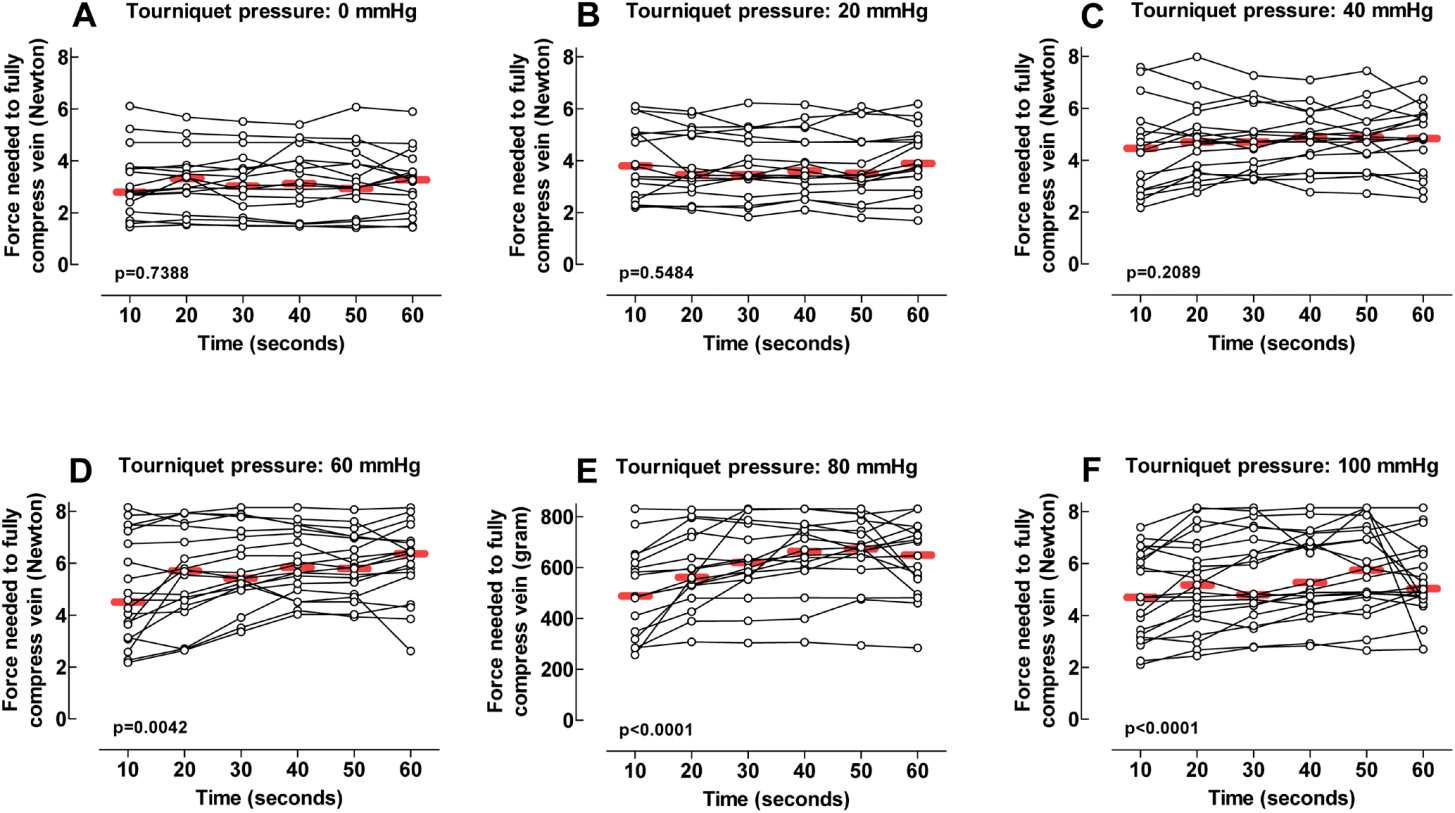
Individual and group median force required to fully compress the vein over 60 s of tourniquet pressure at 0, 20, 40, 60, 80, and 100 mmHg. The red lines indicate the median force required to fully compress the vein at the various time points.

For the force required to fully compress the vein (Figure 3), time‐dependent changes were significant only at pressures of 60 mmHg and above. However, as the first 10 s after tourniquet application were not measured, the initial adaptation phase was not captured. When comparing the median values at each pressure level to those obtained without a tourniquet, all tourniquet pressures showed significantly reduced venous compliance. Similar to the diameter data, the responses varied markedly between participants in both magnitude and timing, demonstrating a high degree of interindividual variability. Individual time‐dependent changes in the force needed to fully compress the veins for all participants are presented in Table S2.

**FIGURE 4:**
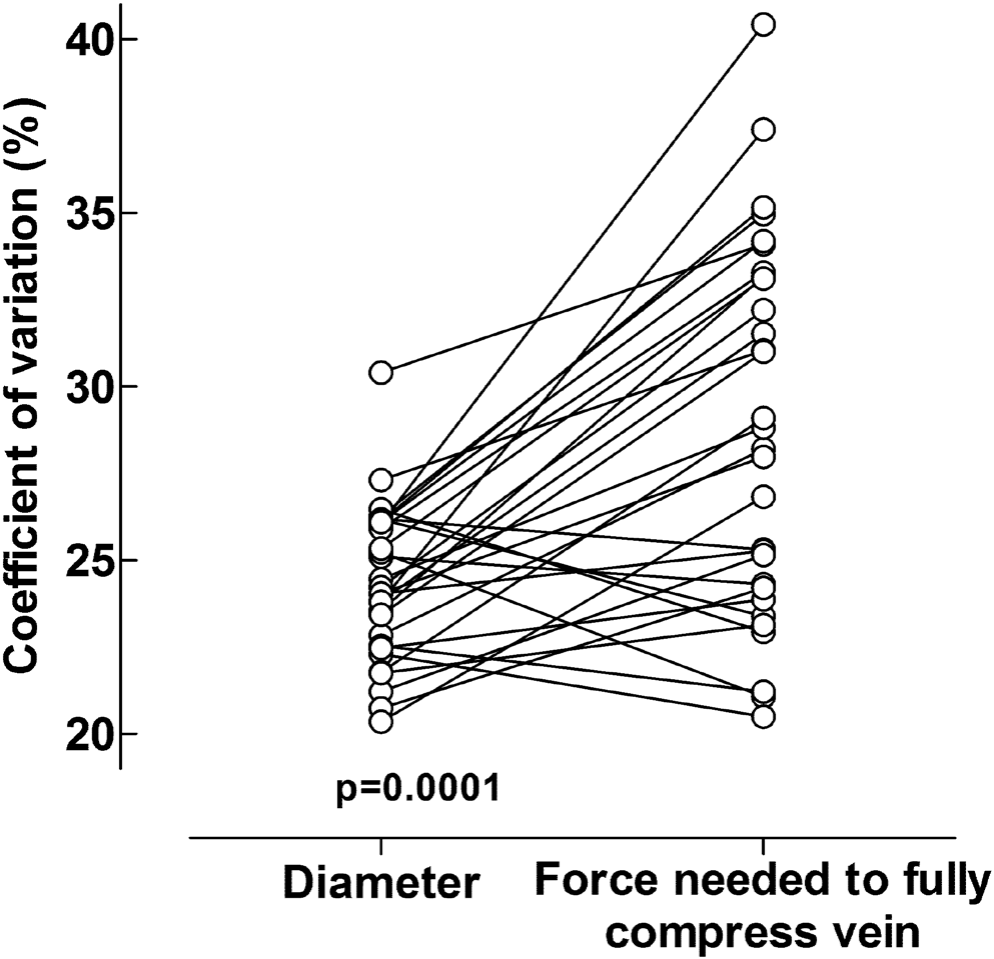
Within‐subject variability in vein diameter and compression force. Each point pair shows the coefficient of variation (%) for repeated measurements of diameter and compression force in individual participants. Lines connect paired values.

Variability expressed as coefficient of variation (%) for repeated measurements of diameter and compression force is presented in Figure 4. The variability in compression force was significantly greater than the variability in vein diameter (*p* = 0.0001; Figure 4).

**FIGURE 5:**
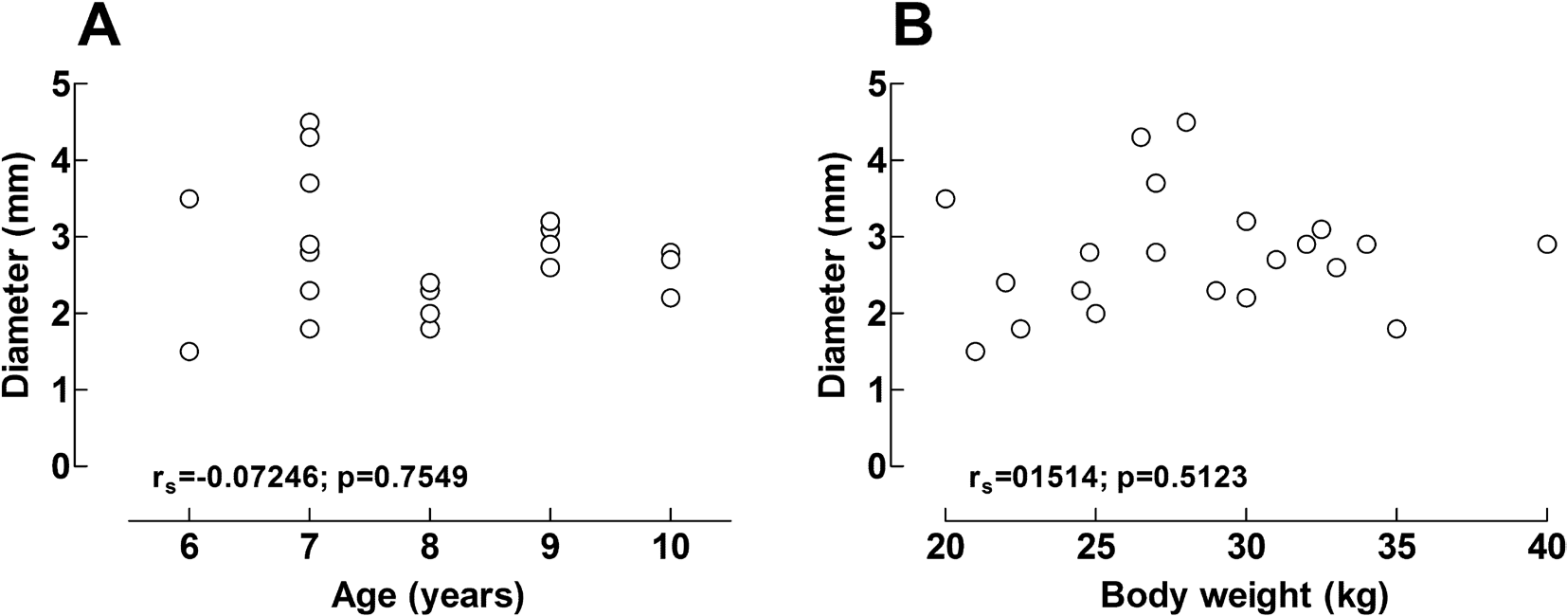
Associations between vein diameter and (A) age and (B) body weight. Individual values are shown.

There was substantial variation in vein diameter among the participants, ranging from approximately 1–5 mm (Figure 5). No correlation was found between vein size and neither age (Figure 5A) nor body weight (Figure 5B).

## 4 Discussion

Anatomical, physiological, and developmental differences make PIVC insertion considerably more complex in children than in adults [9]. One major challenge is the small size of paediatric veins, which makes them difficult to visualise, palpate, and identify as suitable targets for catheterization [1, 9]. Even a marginal increase in vein size can improve the likelihood of successful catheter placement. The fact that a 0.1 mm decrease in catheter size from 0.7 mm (24G) to 0.6 mm (26G) is considered clinically relevant highlights that even minor variations in vein diameter can meaningfully influence the success of PIVC insertion [10]. In addition to their size, paediatric veins are also highly compliant and collapse easily under external pressure, which further complicates PIVC insertion [11]. The high compliance of veins presents notable challenges during PIVC insertion. Instead of being perforated by the cannula, compliant veins are easily compressed by the advancing needle. The resulting indentation in the vein reduces its diameter prior to penetration, increasing the risk that the needle tip will traverse the entire vein rather than remain within the lumen [11, 12, 13]. A successful catheter placement requires the needle tip to remain intraluminal to avoid posterior wall perforation. The risk of posterior wall perforation is pronounced in children due to the combination of small vein size and high venous compliance, which leaves virtually no margin for error during cannulation [13]. Applying a tourniquet is a common technique to dilate veins before PIVC insertion, providing a larger target area for needle placement [6, 7, 14, 15, 16, 17] and reducing venous compliance [6]. In adults, a tourniquet pressure of 60–80 mmHg applied for 30–60 s has been shown to maximise vein dilation [7, 16]. No corresponding studies in children have been conducted, and existing guidelines for PIVC insertion in children are based solely on expert consensus.

The findings of the present study reflect both measurement‐related variability and individual differences in vascular response. Although certain tendencies emerged at the group level, the results indicate that within the age group of 6–10 years, paediatric veins respond individually to external pressure in terms of both magnitude and timing. This likely reflects interindividual differences in the biomechanical properties of superficial veins.

The median values presented in Figures 2 and 3 suggest that the maximum vein diameters and the greatest force required to fully compress the veins occur within the 60‐s measurement period, consistent with the observations reported by Sasaki et al. [7]. However, since Sasaki et al. did not provide individual participant data, it remains unclear whether their participants exhibited a similar degree of interindividual variability as observed in the present study.

There was a substantial variation in vein diameter between the participants (Figure 5), with measurements ranging from approximately 1 to 5 mm, consistent with previous research [18, 19]. Although previous studies have observed some correlation with body weight in older children, there appears to be considerable individual variation in vein size which cannot be explained by factors such as age, weight, or sex [18, 19]. The absence of reliable reference standards for peripheral vein dimensions in children limits the clinical value of these demographic predictors. As a result, ultrasound is required for accurate individual assessment.

The present study did not include any measurements or systematic documentation of participants' subjective experiences of different tourniquet pressures. All pressure levels were tolerated by the participants, but some of the children required mild distraction to remain still. It was evident that higher tourniquet pressures caused increased discomfort, which appeared to intensify over time following tourniquet application. Therefore, from a participant comfort perspective, lower tourniquet pressures and shorter application times are clearly preferable whenever clinically feasible.

Some limitations should be considered when interpreting these findings. Combining ultrasound measurements with intermittent compressions from the transducer increased the measurement uncertainty and likely contributed to the observed variability. These compressions disrupted the continuity of vein visualisation and may also have induced physiological changes affecting vein size.

The order of tourniquet pressures was not randomised, but followed a sequence from lowest to highest. This approach was chosen to allow participants to gradually adapt to the increasing pressure levels, thereby minimising discomfort and promoting better compliance. However, this design carries an inherent risk of order effects, as physiological changes over time might have influenced the measurements.

The choice of diameter as the primary measure of vein size can be debated. However, in clinical practice, the selection of an appropriate size of PIVC is typically based on its diameter in relation to the vein diameter.

Although most target veins were relatively superficial, vein palpability and visibility were not systematically assessed. This limits the ability to relate ultrasound findings to clinical estimations of vein accessibility, which are commonly used in practice when selecting cannulation sites.

## 5 Conclusions

This study demonstrates that tourniquet application produces a marked increase in venous diameter and a corresponding reduction in venous compliance in children. However, there is a substantial interindividual variation in how children's veins respond, both in terms of venous diameter and in terms of compliance. Consequently, neither a specific tourniquet pressure nor a fixed time after application can be recommended as universally applicable to improve the conditions for PIVC insertion in children. Real‐time ultrasound is the only reliable method for assessing the effects of tourniquet use, but its limited availability restricts its clinical use. Future research should therefore focus on developing alternative methods that can provide guidance during PIVC insertion when ultrasound is not accessible.

## Author Contributions

**Karl Rönnegård:** conceptualization, methodology, software, validation, formal analysis, investigation, data curation, writing – original draft, writing – review and editing, project administration, funding acquisition. **Staffan Eksborg:** methodology, software, validation, formal analysis, writing – original draft, writing – review and editing, visualization, supervision. **Pedram Hosseiniakram:** software, validation, investigation, writing – review and editing. **Per‐Arne Lönnqvist:** methodology, validation, writing – review and editing, supervision. **Dmitry Grishenkov:** methodology, validation, resources, writing – review and editing, supervision. **Gunilla Lööf:** conceptualization, methodology, investigation, writing – review and editing, supervision, project administration, funding acquisition.

## Funding

This work was supported by grants from Stiftelsen Solstickan and Stiftelsen Samariten.

## Conflicts of Interest

The authors declare no conflicts of interest.

## Supporting information


**Table S1:** Individual time‐dependent changes in vein diameter. Data from all participants.

**Table S2:** Individual time‐dependent changes in the force required to fully compress the veins. Data from all participants.

## Data Availability

The data that supports the findings of this study are available in the Supporting Information of this article.
